# Accuracy of an artificial intelligence algorithm for detecting moderate-to-severe vertebral compression fractures on abdominal and thoracic computed tomography scans

**DOI:** 10.1590/0100-3984.2023.0102

**Published:** 2024-05-03

**Authors:** Renata Fernandes Batista Pereira, Paulo Victor Partezani Helito, Renata Vidal Leão, Marcelo Bordalo Rodrigues, Marcos Felippe de Paula Correa, Felipe Veiga Rodrigues

**Affiliations:** 1 Hospital Sírio-Libanês, São Paulo, SP, Brazil; 2 Instituto de Radiologia do Hospital das Clínicas da Faculdade de Medicina da Universidade de São Paulo (InRad/HC-FMUSP), São Paulo, SP, Brazil.; 3 Department of Radiology, Aspetar Qatar Orthopaedic and Sports Medicine Hospital. Doha, Qatar

**Keywords:** Fractures, compression/diagnostic imaging, Spinal fractures/diagnostic imaging, Lumbar vertebrae/diagnostic imaging, Thoracic vertebrae/diagnostic imaging, Osteoporosis, Artificial intelligence, Fraturas por compressão/diagnóstico por imagem, Fraturas da coluna vertebral/diagnóstico por imagem, Vértebras lombares/diagnóstico por imagem, Vértebras torácicas/diagnóstico por imagem, Osteoporose, Inteligência artificial

## Abstract

**Objective:**

To describe the accuracy of HealthVCF, a software product that uses artificial intelligence, in the detection of incidental moderate-to-severe vertebral compression fractures (VCFs) on chest and abdominal computed tomography scans.

**Materials and Methods:**

We included a consecutive sample of 899 chest and abdominal computed tomography scans of patients 51–99 years of age. Scans were retrospectively evaluated by the software and by two specialists in musculoskeletal imaging for the presence of VCFs with vertebral body height loss > 25%. We compared the software analysis with that of a general radiologist, using the evaluation of the two specialists as the reference.

**Results:**

The software showed a diagnostic accuracy of 89.6% (95% CI: 87.4–91.5%) for moderate-to-severe VCFs, with a sensitivity of 73.8%, a specificity of 92.7%, and a negative predictive value of 94.8%. Among the 145 positive scans detected by the software, the general radiologist failed to report the fractures in 62 (42.8%), and the algorithm detected additional fractures in 38 of those scans.

**Conclusion:**

The software has good accuracy for the detection of moderate-to-severe VCFs, with high specificity, and can increase the opportunistic detection rate of VCFs by radiologists who do not specialize in musculoskeletal imaging.

## Abstract (pt)

**Objetivo:**

Descrever a acurácia do *software* HealthVCF na detecção incidental de fraturas compressivas de corpos vertebrais moderadas a graves em exames de tomografia computadorizada do tórax e abdome.

**Materiais e Métodos:**

Foram incluídos 899 exames consecutivos de pacientes com idades entre 51 e 99 anos. As imagens foram retrospectivamente avaliadas pelo *software* e por dois radiologistas especializados em musculoesquelético que investigaram fraturas compressivas de corpos vertebrais com perda da altura somática > 25%. A análise comparativa foi realizada entre o *software* e um radiologista geral, usando a avaliação do especialista como referência.

**Resultados:**

O *software* apresentou uma acurácia de 89,6% (IC 95%: 87,4–91,5%) para fraturas compressivas moderadas a graves, com sensibilidade de 73,8%, especificidade de 92,7% e valor preditivo negativo de 94,8%. Entre as 145 tomografias positivas detectadas pelo *software,* o radiologista geral deixou de relatar as fraturas em 62 (42,8%) e o algoritmo detectou fraturas adicionais em 38 dessas tomografias.

**Conclusão:**

O *software* possui boa acurácia na detecção de fraturas compressivas moderadas a graves, com alta especificidade, podendo aumentar a taxa de detecção oportunística dessas fraturas por radiologistas não especializados em musculoesquelético.

## INTRODUCTION

Osteoporosis is defined as a skeletal disease characterized by compromised bone mass, strength, and microarchitecture, which increases the propensity for fragility fractures. It represents a prevalent public health problem in the population over 50 years of age and disproportionately affects women, being present in approximately 40% of all postmenopausal White women^(1, 2)^. It is estimated that there are nine million osteoporotic fractures per year worldwide, which has significant physical, psychosocial, and financial impacts on patients and society, as well as being associated with high rates of morbidity and mortality^(3,4,5)^.

The vertebral body is the site most affected by fractures, especially in the middle segment of the thoracic spine and at the thoracolumbar junction^(6)^, and fragility fractures often represent the first opportunity for osteoporosis care. Although some vertebral compression fractures (VCFs) have a significant clinical presentation, most are oligosymptomatic and are often underdiagnosed or diagnosed incidentally on imaging tests^(7,8,9,10)^. Early detection of VCFs is also important because partial compression of one vertebra increases the risk of progressive compression and subsequent fractures—by 5.0—12.6 times in other vertebrae and by 2.3—3.4 times in the hip^(11)^. Therefore, whether symptomatic or asymptomatic, compression fractures have significant consequences for the patient due to the increased risk of new fractures and the high rates of morbidity and mortality^(3,1,12)^.

Patients in the age group at high risk for VCFs frequently undergo imaging tests that encompass the vertebral column, providing an opportunity to screen for oligosymptomatic fractures. On computed tomography (CT) scans of the chest and abdomen, VCFs are often underdiagnosed, rarely being referenced in the corresponding radiology reports^(10)^. In this context, the application of automated VCF detection software might increase radiological accuracy for fracture detection on scans that do not target the spine but include it in the imaging, facilitating early incidental diagnosis of osteoporosis and opening possibilities for earlier interventions.

Although some studies have described the accuracy of automated fracture detection software^(13,14)^, the variability between populations and algorithms used must be considered when the results are interpreted. Our study aims to describe the accuracy of an artificial intelligence (AI)-based software product designated HealthVCF (Zebra Medical Vision Ltd., Shefayim, Israel) for incidental fracture detection on chest and abdominal CT scans, using consensual assessments by radiologists specialized in musculoskeletal imaging as the reference standard.

## MATERIALS AND METHODS

### Study population

After receiving approval from the local institutional review board (Reference no. 19292619.9.0000.5461), a cross-sectional retrospective cohort study was conducted. Because of the retrospective nature of the study, the requirement for informed consent was waived. The study utilized a consecutive sample of 964 CT scans, which were not ordered specifically for assessment of the spine but included it within the field-of-view. These consisted of chest and abdominal CT scans performed in patients between 51 and 99 years of age, for various clinical indications, over a one-year period in the radiology department of our hospital. The reports were provided by general radiologists who were not specialists in musculoskeletal imaging.

Of the 964 scans selected for evaluation, 54 were excluded because they could not be evaluated by the algorithm: 37 because they could not be analyzed (unavailable axial series in 24 and incomplete examinations in 13); and 17 because of failure during the analysis (the spine could not be segmented, mainly because of distortion of the vertebral body or the presence of metallic objects). Subsequently, 11 more scans were excluded because of the presence of metastases (pathological fractures). Therefore, the final sample comprised 899 valid scans, of which 493 were chest CT scans (440 conventional CT scans and 53 CT angiograms) and 406 were abdominal scans (conventional CT scans). Intravenous injection of iodinated contrast was not considered a criterion for inclusion or exclusion.

All CT examinations were performed with volumetric acquisition in the axial plane, at a slice thickness of 1.0mm for the chest CT scans and 3.0-mm for the abdominal CT scans, in multidetector CT scanners (Siemens Health-ineers, Erlangen, Germany). The scanning parameters were a tube voltage of 120 kVp and a tube current adjusted from 84 mAs to 130 mAs (mean, 107 mAs). In addition, 3 mm-thick sagittal reconstructions were available for the abdominal CT scans. Sagittal reconstructions of the thoracic and lumbar spine were routinely performed.

### Image analysis

**Figure 1:**
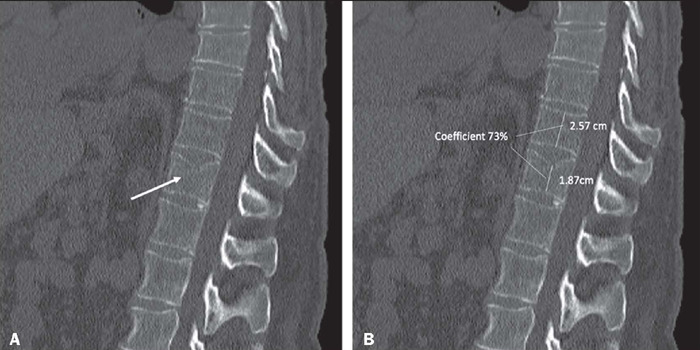
Clinically relevant fracture of the T8 upper vertebral plateau in a 56-year-old patient, in a case detected by the algorithm. **A, B:** Sagittal reconstruction of a chest CT showing the T8 fracture (arrow in **A**) and the measurement of the vertebral body height loss, which was found to be 27% with the measurement tool (**B**).

Two radiologists specialized in musculoskeletal imaging with four and ten years of experience, respectively, analyzed the images and the corresponding reports using software for picture archiving and communication system (PACS) evaluation (Kodak DirectView PACS System 5, version 5.2; Carestream Health, Rochester, NY, USA), to identify VCFs by visual and quantitative inspection. The two specialists reviewed the CT scans independently, and disagreements were resolved by consensus. They classified the fractures by location and by the percentage of vertebral height lost. The percentage of height lost was determined manually by comparing the central portion of the compressed vertebra with the adjacent non-compressed vertebra by using the standard measuring tool of the PACS system in the sagittal images (Figure 1). In cases with four or more fractures, only the three fractures with the greatest vertebral body height loss were evaluated.

Fracture severity was determined according to the semiquantitative method devised by Genant et al.^(15)^: grade 0, normal; grade 1, mild deformity (≥ 20% and < 25% reduction in anterior, middle, or posterior height); grade 2, moderate deformity (≥ 25 and < 40% reduction in the height of any portion); and grade 3, severe deformity (> 40% reduction in the height of any portion).

The tests were anonymized and sent separately, in Digital Imaging and Communications in Medicine format, for independent evaluation by the fracture-identifying component of HealthVCF, which was approved by the U.S. Food and Drug Administration in 2020. Using deep neural network technology, the software extracts a sagittal section of the spinal mid-plane and identifies vertebral fractures by using a combination of convolutional and recurrent neural network technology. The evaluation performed by the software was dichotomous (presence/absence of at least one VCF). For positive and negative cases, respectively, the software displayed the messages “At least one vertebral compression fracture has been detected” and “No fracture was detected”^(16)^. To automatically detect VCFs, the algorithm consists of three processes. First, the spine is segmented and the sagittal segments are extracted. Binary classification of the segments is then performed by using a convolutional neural network. Finally, a recurrent neural network is used in order to predict whether a VCF is present in the segment series.

We also evaluated the previously issued final radiology report to determine whether the general radiologist (not a specialist in musculoskeletal radiology) had described the fracture or made any reference to a previous radiological description of the fracture. This evaluation aimed to assess the number of fractures not prospectively reported by the general radiologist and compare it with the number identified by the software.

### Data analysis

Fractures were considered moderate when there was vertebral body height loss ≥ 25% (Genant grade 2). The consensual analysis of the musculoskeletal radiologists was used as the reference standard for the detection and classification of fractures. Data from the reference standard were tabulated and subsequently compared with the HealthVCF software data to determine the diagnostic accuracy of the latter.

### Statistical analysis

Statistical analysis was performed with the IBM SPSS Statistics software package, version 22.0 (IBM Corp., Armonk, NY, USA). The performance of the HealthVCF algorithm in identifying clinically relevant VCFs was analyzed with the chi-square test. We assessed the accuracy, sensitivity, specificity, positive predictive value, and negative predictive value of the algorithm. A 95% confidence interval was established, and a significance level of 0.05 was employed.

## RESULTS

The patients ranged in age from 51 to 99 years, with a mean age of 70.2 years (Table 1). Although most of the scans (51.8%) were performed in patients between 60 and 79 years of age, nearly half of the fractures (47.0%) were diagnosed in those between 80 and 99 years of age. We performed no independent evaluations based on sex, previous diagnosis of osteoporosis, or the presence of other comorbidities.

**Table 1:** Demographic and fracture characteristics.

Characteristic	(N = 899)
Age (years), n (%)	
50–59	208 (22.9)
60–69	251 (27.6)
70–79	219 (24.1)
80–89	166 (18.3)
90–99	64 (7.0)
Type of scan, n (%)	
Chest CT angiography	55 (6.1)
Abdominal CT	407 (44.8)
Chest CT	446 (49.1)
Clinically relevant VCF, n (%)	
No	754 (83.0)
Yes	145 (17.0)
Number of VCFs, n (%)	
None	754 (79.2)
1	110 (12.1)
2	37 (4.1)
3	42 (4.6)
Algorithm result, n (%)	
No VCFs	792 (88.0)
At least one VCF	107 (12.0)

Among the 899 scans selected, the musculoskeletal specialists detected fractures in 195 (21.6%) and classified the fractures as moderate-to-severe (vertebral height loss ≥ 25%) in 145 (16.1%). Fracture of a single vertebra was the most common finding (in 58.2%), followed by three or more fractures (in 22.2%) and two fractures (in 19.5%).

In the positive scans, we evaluated a total of 310 fractures, which were distributed from the T1 to L4 vertebral bodies, with the majority being located at the thoracolumbar junction, mainly affecting the T12 vertebral body (in 14.4%). Fractures in the upper thoracic (T1–T4) and lower lumbar (L3 and L4) segments were uncommon (seen in only 12.6% and 5.8%, respectively).

Table 2 shows the HealthVCF algorithm detection rates, and Table 3 shows the comparison between the algorithm and the reference standard. The algorithm identified clinically relevant VCFs in 107 of the 145 positive scans, translating to an accuracy of 89.6% (95% CI: 87.4–91.5%). The algorithm had a sensitivity of 73.8% (95% CI: 65.7–80.5%) and a specificity of 92.7% (95% CI: 90.5–94.4%), with a positive predictive value of 66.0% (95% CI: 58.1–73.1%), and a negative predictive value of 94.8% (95% CI: 92.9–96.2%).

**Table 2:** Algorithm detection rate by scan type.

Type of scan	Moderate-to-severe VCF		Total (n)	Sensitivity (%)	Specificity (%)
	No (n)	Yes (n)			
Chest CT angiography	Algorithm result, n				66.6	100
No VCFs detected	47	2	49		
VCF(s) detected	0	4	4		
Total	47	6	53		
Abdominal CT					
Algorithm result, n				73.9	95.0
No VCFs detected	342	12	354		
VCF(s) detected	18	34	52		
Total	360	46	406		
Chest CT					
Algorithm result, n				80.6	91.3
No VCFs detected	317	18	335		
VCF(s) detected	30	75	111		
Total	347	93	440		
Total					
Algorithm result, n				73.8	92.7
No VCFs detected	699	38	737		
VCF(s) detected	55	107	162		
Total	754	145	899		

**Table 3:** Confusion matrix of VCF detection on CT scans. Actual VCFs detected by the reference standard versus algorithm-predicted VCFs.

Algorithm result	Actual fractures detected by the reference standard		
	Moderate-to-severe VCF(s)	No VCFs	Total
Positive test	55	107	162
Negative test	699	38	737
Total	754	145	899

The general radiologist identified and reported moderate-to-severe VCFs in 65 (44.8%) of the 145 positive scans, compared with 107 (73.8%) for the algorithm. Of the 80 scans in which fractures were not reported by the general radiologist, 18 (22.5%) had actually been described in a spine examination conducted within the last six months. Therefore, there were in fact 62 scans in which a general radiologist did not prospectively report fractures. Among those 62 scans, the algorithm successfully detected 38 in which there were unreported fractures, resulting in an additional detection rate of 61.2%.

## DISCUSSION

We evaluated the diagnostic accuracy of AI-based software designed for automated detection of VCFs in chest and abdominal CT scans. The performance of the software was compared with assessments made by general radiologists and with the consensus assessment of two musculoskeletal radiologists, which was used as the reference standard. The results show that the software achieved good overall accuracy (89.6%), excellent specificity (92.7%) and moderate sensitivity (73.8%). Despite the moderate sensitivity, the HealthVCF software should be considered a promising method for opportunistic diagnosis of VCF.

When comparing the HealthVCF algorithm with others employed in the evaluation of thoracolumbar spine fractures on CT images, we noted that its sensitivity (73.8%) was considerably lower than the 91.0–95.7% reported for similar algorithms^(13,14)^. That difference in sensitivity holds significance for an opportunistic diagnostic test. However, it is essential to highlight that we applied a more specific threshold by selecting as positive only those scans that showed a moderate-to-severe VCF (defined as a loss of vertebral body height ≥ 25%), which provided a specificity of 92.7%, considerably higher than the 77.3% reported for the algorithm evaluated by Burns et al.^(13)^.

Although the general radiologists had a relatively high (44.8%) VCF detection rate in the initial reports, the AI algorithm was able to detect additional fractures in more than half of the scans in which fractures had not been initially reported. In an analysis based on the number needed to harm, we estimated that the use of the algorithm could modify the diagnostic outcome in one out of every 23.6 scans. Given the unexpected nature of a finding of osteoporotic vertebral fractures on routine scans of the chest and abdomen, any potential increase in the fracture detection rate in at-risk populations is beneficial, especially if it does not require any additional procedure or examination.

Early diagnosis of VCFs and osteoporosis is essential for effective case management and for reducing the significant economic burden on health systems. Joestl et al.^(17)^ studied a population of 694 patients with VCFs, 45% of whom required hospitalization and extensive rehabilitation with physical therapy or the use of orthotics, resulting in a high estimated cost per patient. Therefore, using software that increases the rate of VCF detection may lead to cost savings by enabling the early diagnosis and treatment of osteoporosis, as well as by preventing new fractures and reducing morbidity.

Carberry et al.^(18)^ and Bartalena et al.^(10)^ reported VCF prevalence rates of 4.8% and 9.5%, with VCF detection rates of 16.0% and 14.6%, respectively, whereas the rates derived from the initial radiology reports by general radiologists in the present study were 16.1% for VCF prevalence and 44.8% for VCF detection. The higher prevalence of fractures in the present study might be due to the broader age range of the patients included in the samples studied by those two groups of authors (19–94 years and 20–88 years, respectively), whereas we evaluated only patients ≥ 51 years of age. It is well known that VCFs are more common in older individuals, particularly in postmenopausal women, which underscores the need for opportunistic screening in such age groups, to improve the detection of fractures and osteoporosis. Our higher rate of fracture detection could be attributed to the routine use of sagittal reconstructions of chest and abdominal CT scans and the use of multiplanar reconstruction in the PACS system. Axial images alone are inadequate for VCF detection, with a reported detection rate of only 35%^(19)^. Therefore, the evaluation of sagittal images is usually essential for the diagnosis and classification of fractures^(20)^.

This study has some limitations. First, the exclusion of some cases due to the primary failure of evaluation by the algorithm demonstrates an intrinsic limitation of the method that can lead to a selection bias, which could skew the sensitivity and specificity estimates. In addition, the software did not localize the fractures, rather serving to alert the radiologist regarding the presence of a fracture in the scan, which limits the benefits of its clinical usage. Furthermore, the software was tested for the detection of only those VCFs with vertebral body height loss ≥ 25% (i.e., moderate-to-severe fractures), which could have increased the specificity for the detection of such fractures. However, mild (Genant grade 1) fractures may also have clinical relevance because they can provide an early diagnosis of osteoporosis, thus enabling the treatment and prevention of new fractures, and should therefore be addressed.

The data presented support the hypothesis that the use of the AI-based software HealthVCF could increase the rate of VCF detection by general radiologists on CT examinations of the chest and abdomen. Such software programs have been constantly elaborated upon and improved, and prospective studies evaluating clinical outcomes are needed in order to validate their use.
